# Original Contributions Concerning the Glandular Structures Appertaining to the Human Eye and Its Appendages

**Published:** 1902-12

**Authors:** 


**Original Contributions concerning the Glandular Structures appertaining to the Human Eye and its Appendages.** By Adolf Alt, M.D, Pp. 23, and xxxvi. plates. St. Louis: *American Journal of Ophthalmology.* 1900.

No excuse for the publication of such admirable work by Professor Alt is needed; and as he points out that the old operation of removal of the lachrymal glands for incurable lachrymation has now become a legitimate surgical procedure, his investigations become of practical interest. Besides the orbital lachrymal gland, he describes most minutely the palpebral lachrymal gland separated from the former by the levator palpebræ superioris and Müller's non-striated muscle. This little gland, or group of glands, extends a variable distance towards the nasal side of the upper lid, and round the external canthus into the lower lid. These glands do not, as is usually described, open into the duct of the orbital gland, but into one or more ducts for each part of the group. He also finds that there is usually a pair of very small similar glands, one above and one below the caruncle. These facts, and numerous other minute details relating to the glands of the lid margin, are substantiated by sixty-eight photomicrographs, which are in every instance unusually good.

